# Eicosapentaenoic Acid (EPA) and Docosahexaenoic Acid (DHA): A Targeted Antioxidant Strategy to Counter Oxidative Stress in Retinopathy

**DOI:** 10.3390/antiox14010006

**Published:** 2024-12-24

**Authors:** Marco Zeppieri, Caterina Gagliano, Fabiana D’Esposito, Mutali Musa, Irene Gattazzo, Maria Sole Zanella, Federico Bernardo Rossi, Alessandro Galan, Silvia Babighian

**Affiliations:** 1 Department of Ophthalmology, University Hospital of Udine, 33100 Udine, Italy; 2 Department of Medicine and Surgery, University of Enna “Kore”, 94100 Enna, Italy; 3 Mediterranean Foundation “G.B. Morgagni”, 95125 Catania, Italy; 4 Imperial College Ophthalmic Research Group (ICORG) Unit, Imperial College, 153–173 Marylebone Rd, London NW1 5QH, UK; 5 Department of Neurosciences, Reproductive Sciences and Dentistry, University of Naples Federico II, 80131 Napoli, Italy; 6 Department of Optometry, University of Benin, Benin City 300238, Edo State, Nigeria; 7 Africa Eye Laser Centre, Benin City 300105, Edo State, Nigeria; 8 Department of Ophthalmology, Ospedale Sant’Antonio, Azienda Ospedaliera, 35127 Padova, Italysilvia.babighian@aopd.veneto.it (S.B.); 9 Department of Translational Medicine, University of Ferrara, 44121 Ferrara, Italy; 10 PhD Program, Arterial Hypertension and Vascular Biology ARHYVAB, University of Padua, 35121 Padova, Italy

**Keywords:** omega-3 fatty acids, diabetic retinopathy, age-related macular degeneration

## Abstract

Omega-3 fatty acids are critical components of cell membranes, including those in the retina. Specifically, eicosapentaenoic acid (EPA) and docosahexaenoic acid (DHA) are the primary omega-3 fatty acids that have been studied for their potential benefits in retinal health, preventing the progression of retinopathy. Several studies have shown that a higher intake of omega-3 fatty acids is associated with a lower risk of developing diabetic retinopathy and age-related macular degeneration (AMD). Reviewing clinical trials and observational studies that support the protective role of omega-3s in retinal disorders is essential. This comprehensive review aims to evaluate the current literature on the role of omega-3 fatty acids, exploring their mechanisms of action and anti-inflammatory, anti-angiogenic, and neuroprotective roles in the retina. Omega-3s have been shown to inhibit abnormal blood vessel growth in the retina, which is a significant factor in proliferative diabetic retinopathy and neovascular AMD. Furthermore, omega-3 fatty acids are often studied with other nutrients, such as lutein, zeaxanthin, and vitamins, for their synergistic effects on retinal health. Reviewing these combinations can help understand how omega-3s can be part of a comprehensive approach to preventing or treating retinopathies, especially in diabetic patients. This review emphasizes the preventive function of EPA and DHA in alleviating oxidative stress-related damage in retinal diseases, concentrating on their antioxidative mechanisms.

## 1. Introduction

Omega-3 polyunsaturated fatty acids (PUFA) play a central role in several retinal mechanisms, including antioxidative processes. Since oxidative-induced damage is one of the mechanisms that lead to the multifactorial pathological process of diabetic retinopathy, understanding their role could enhance their potential use in preventing several retinal pathologies [1]. Most PUFAs are esterified to phospholipids [2]. Docosahexaenoic acid 22:6 omega-3 (DHA) is the most abundant retinal omega-3 component and is produced by RPE and retinal cells [3]. Moreover, RPE delivers DHA from the systemic microcirculation to the photoreceptors [4]. Oxidative stress is a significant contributor to the development of retinal diseases, including diabetic retinopathy and age-related macular degeneration (AMD). This review assesses the function of omega-3 fatty acids, specifically EPA and DHA, in mitigating oxidative stress and their potential as therapeutic agents for retinal health.

DHA presence in cellular membranes is essential for rhodopsin conformational changes (and consequently to the visual cascade) through its contribution to cellular membrane fluidity; indeed, it contributes to maintain cellular structure [5,6,7]. Its anti-inflammatory properties have been explored under different conditions, including hyperlipidemia, an independent risk factor for retinal inflammation status promoted by MCP-1, TNF-alfa, and IL-1beta [8]. It influences many cellular functions as ion channels, receptor activation, and the induction of signaling pathways [5]. DHA contributes to the downregulation of pro-inflammatory genes and balances lipid levels, as well as through triglycerides level decrease [7,8,9]. A loss of balance between lipids seems to lead to photoreceptor degradation [7].

DHA contributes to anti-inflammatory mechanisms, especially under oxidative stress, as well as through the derivation of lipid mediators like neuroprotectin D-1, which provides retinal protection through the prevention of epithelial and neuronal glial cells from apoptosis [1,10]. DHA protects ganglion cells from oxidative damage and prevents oxidative-induced apoptosis of photoreceptors [3]; additionally, it leads to the production of a lipid mediator called 14S,21R-diDHA, which contributes to multiple antidiabetic actions, including HGF generation, IL-10 increase, IL-1 beta decrease, pericyte density increase, wound healing, and the prevention of mesenchymal stem cells (MSCs) from apoptosis, mainly stimulating MSC paracrine functions [10]. Maresins also contribute to the augmentation of pericyte density, the inhibition of ferroptosis, and the maintenance of photoreceptor structure [10,11].

EPA is another omega-3 component that plays a crucial role in protecting retinal tissue from oxidative damage, not only through its direct effect on rescuing photoreceptor from apoptosis and enhancing photoreceptor differentiation but also through its elongation and desaturation to DHA by retinal neurons [3,4]. Resolvin E1 is a molecule produced by EPA and DHA with anti-inflammatory properties [1,10]. EPA has a role in the preservation of photoreceptor mitochondrial membrane potential [3]. Its anti-inflammatory actions, as well as DHA, are mainly mediated by the inhibition of I-CAM, MCP-1, VEGF, IL-6, and IL-18 [12]. Furthermore, a recent study evaluated the effects of omega-3 PUFAs on lipofuscin granules, demonstrating a reduced retinal accumulation [12]. In conclusion, PUFAs’ anti-inflammatory mechanisms are mainly conducted through the inhibition of inflammatory cytokines or adhesion molecules, chemotaxis, and complement activation [4,12].

Additionally, n3-PUFAs reduce free radical generation, and in particular, some studies conducted in diabetic patients demonstrated that EPA supplementation leads to glutathione peroxidase and superoxide dismutase increase [4]. PUFAs are vulnerable to oxidative processes, giving rise to a fragment that completes several cycles and has been proposed as an active contributor to age-related macular disease (AMD) [3]. Although several antioxidant molecules (e.g., vitamin E) normally protect tissues from oxidant damages, retinal pathologies can compromise such physiological defense [13]. For this reason, the deuterated form of DHA at the bis-allylic positions (D-DHA) has been proposed as a dietary supplementation to provide a more resistant PUFA; additionally, D-DHA has a great distribution in neurological and retinal tissue [13].

DHA and EPA anti-inflammatory activities are mediated not only directly but also through the competition with arachidonic acid (AA) for the production of mediators in membranes phospholipids, causing a reduction in PGD and PGE production [12]. An increased omega-6/omega-3 ratio is related to a major inflammatory microenvironment, which could lead to AMD [14].

Schuchardt et al. demonstrated that omega-3 is capable of transforming leukocytes properties, reducing their stiffness, especially under stressing conditions; as a result, omega-3 PUFAs seem to optimize cellular response to inflammation and infection [15]. DHA and EPA demonstrated some antithrombotic effects on the systemic circulation that could also be exerted on the choroid [7]. Finally, omega-3 increases caveolin1 expression [1].

## 2. Clinical Evidence

While in the past, research on the action of omega-3 polyunsaturated fatty acids (DHA and EPA) in the retina has mostly been focused on the animal model, in the last decade. a great deal of clinical and randomized studies have emerged, bringing novel solid evidence and adding clinical data to the field. Several randomized clinical trials aimed at evaluating the effect of DHA and/or EPA in the context of the prevention of the occurrence or delay of the progression of specific retinal diseases, which were in fact conducted in large cohorts of patients.

### 2.1. Age-Related Macular Degeneration: Risk of Progression to Advanced Disease Andnew Presentation

The Age-Related Eye Disease Study 2 (AREDS2) was developed to expand upon the findings of the original AREDS study, focusing on the impact of additional nutrients, specifically DHA and EPA, as daily supplements to the original AREDS formulation, which included vitamin C, vitamin E, beta carotene, zinc, and copper, with the objective of delaying the advancement of age-related macular degeneration. From 2006 to 2012, 4203 patients aged 50 to 85 years, identified as being at high risk for advancing to advanced AMD, were initially recruited from 82 clinical sites. The designation of high risk for progression to advanced AMD includes the presence of either bilateral large drusen or large drusen in one eye alongside advanced AMD in the contralateral eye. Advanced AMD is characterized by central geographic atrophy or retinal indicators of choroidal neovascularization identified through the central grading of stereoscopic fundus photographs or a documented history of treatment for advanced AMD subsequent to study enrollment.

In this randomized, double-blind clinical trial, participants were allocated in a 1:1:1:1 ratio to one of four groups, each receiving different additions to the original AREDS formula: lutein (10 mg) + zeaxanthin (2 mg); DHA (350 mg) + EPA (650 mg); a combination of both; and a placebo (the original AREDS formula). Upon the study’s conclusion in 2013, with a median follow-up duration of 5 years, a total of 1608 participants had encountered at least one advanced AMD episode; no statistically significant adverse effects were observed, nor were there reductions in the progression to AMD or enhancements in visual acuity associated with the EPA + DHA treatment in primary, secondary, and subgroup analyses [16].

Consequent to these findings, the incorporation of DHA and EPA was terminated and excluded from the recently launched AREDS2 supplements; moreover, data concerning the decade-long risk of developing late age-related AMD from the most recent study reports, derived from the 5-year epidemiological follow-up of participants post-trial, regarding the long-term effects of DHA and EPA are consequently absent [17].

The VITAL-AMD study, conceived as an ancillary prespecified investigation within the Vitamin D and Omega-3 Trial (VITAL), is a double-blind, placebo-controlled, randomized clinical trial assessing the effects of vitamin D and omega-3 supplementation (1 g of Omacor Fish oil, containing EPA and DHA in a 1.2:1 ratio) administered both concurrently and separately, compared to placebo for the primary prevention of cancer and cardiovascular disease. The report on the VITAL-AMD study, which examined the incidence of age-related macular degeneration (AMD) in the cohort of 25,871 enrolled patients, yielded noteworthy findings [18].

Over a median duration of 5.3 years, including therapy and follow-up, 324 patients encountered AMD-related events (285 new incidences of AMD and 39 progressions to advanced AMD among those with the illness at baseline). Upon evaluating the separate elements of the primary endpoint (incident AMD and progression of AMD), no meaningful impact of any intervention could be determined. Subsequent to that, a series of clinical trials assessing the potential beneficial effects of DHA and EPA have been published. Among these, a multicenter, randomized, observer-blinded study was conducted involving 109 patients aged 50 years or older, diagnosed with unilateral exudative (wet) AMD. Participants were randomized in a 1:1 ratio to receive either the original AREDS formulation (placebo group) or a supplement of the original AREDS, excluding beta-carotene, along with copper, DHA, lutein, zeaxanthin, resveratrol, and hydroxytyrosol (intervention group).

At the 12-month follow-up, the intervention demonstrated no significant differential impact on visual acuity. However, a noteworthy effect was observed in the reduction in inflammatory cytokines (interferon-gamma, IL-1ß, and tumor necrosis factor alpha) and in the enhancement of the plasma fatty acid profile, characterized by a greater increase in DHA, total n-3 PUFAs, and LCPUFAs, alongside a greater decrease in total n-6 PUFAs, n-6 LCPUFAs, and the n-6/n-3 PUFA and LCPUFA ratios. Additionally, no differences in safety outcomes were reported.

The Nutritional AMD Treatment-2 (NAT-2) Randomized Trial was structured as a single-center, double-blind, prospective, parallel comparative study, aimed at assessing the dynamic remodeling of drusen in patients with unilateral neovascular AMD, who were administered either a placebo or oral DHA (840 mg/day) over a duration of three years. In a cohort of 300 patients aged 55 to 85 years, drusen progression data were evaluated in 167 individuals who did not develop choroidal neovascularization (CNV); no significant correlation between drusen remodeling results and DHA integration was seen [19].

The LUTEGA project was a double-blind, placebo-controlled, randomized clinical trial, including patients with non-exudative AMD, aimed at assessing the impact of lutein, zeaxanthin, and omega-3 PUFAs on macular pigment optical density (MPOD) evaluated using a modified fundus camera. A total of 145 AMD patients were randomized to receive either a placebo or a single or double dose of the following supplements: Lutein (10 mg), Zeaxanthin (1 mg), DHA (100 mg), and EPA (30 mg). Following one year of intervention, a notable increase in MPOD was observed, correlating with an enhancement in the best corrected visual acuity (BCVA) in the intervention groups relative to the placebo group; however, no significant difference was noted between the two groups with varying dosages [20].

Although the majority of clinical trials have concentrated on the impact of omega-3 PUFAs in hindering or averting the advancement of AMD, certain research has explored their potential in preventing or postponing the onset of the disease in healthy individuals. A report examining the retinal functional outcomes in the elderly population. The n-3 Long-Chain Polyunsaturated Acid (OPAL) study, a multicenter, double-masked, randomized trial, aimed to assess the impact of DHA and EPA on electroretinography parameters (slope of a wave in response to bright flash and amplitude of the b wave in response to dim flash), rod and cone function, color vision, and ocular health, yielded notable data [21].

A total of 53 patients, aged 70 to 79 years, without age-related macular degeneration (AMD) and diabetes, were randomized to receive either a placebo of 0.7 g olive oil or capsules containing 0.7 g of n-3 long-chain polyunsaturated fatty acids (0.2 g EPA + 0.5 g DHA). No significant changes in the primary or secondary outcomes were recorded at the 24-month review; however, a slight yet continuous tendency toward increased rod amplitudes was suggested.

### 2.2. Diabetic Retinopathy

Limited clinical investigations have assessed the potential impact of DHA and EPA especially regarding diabetes and the progression of diabetic retinopathy. ASCEND-EYE was conceived as a substudy of the ASCEND study (A Study of Cardiovascular Events in Diabetes), a double-blind, placebo-controlled trial, intended to assess the beneficial effects of omega-3 fatty acids on severe cardiovascular outcomes in diabetic patients aged 40 and above, specifically focusing on ocular outcomes within the trial population [22].

In the original study involving a total cohort of 15,480 adults with diabetes, who were randomized to receive either a daily placebo or capsules containing 1 g of omega-3 fatty acids (comprising 460 mg of EPA and 380 mg of DHA), 7360 participants residing in England and Wales, who provided consent and attended the scheduled visits of the Diabetic Eye Screening Program, were included in this substudy. Following an average follow-up duration of 6.5 years, 548 individuals in the intervention cohort and 513 in the placebo cohort experienced a referable disease event (characterized as a composite of referable retinopathy with pre-proliferative or proliferative lesions or maculopathy), indicating no significant disparity between the placebo and active groups.

Similarly, secondary and tertiary outcomes collectively demonstrated no significant differences between the two groups, encompassing the time to first occurrence of referable disease post-randomization, progression in retinopathy grade across both eyes, the incidence of diabetic maculopathy, the deterioration of visual acuity, and sensitivity analyses regarding the final and highest recorded retinopathy grades. Conceived as a pre-specified prospective study within the Prevención con Dieta Mediterrànea PREDIMED randomized clinical trial, this investigation was specifically aimed at assessing the occurrence of sight-threatening retinopathy in patients older than 55 years with type 2 diabetes. Patients enrolled in the original study underwent baseline and yearly dietary intake assessment, were thoroughly instructed on diet adherence, and randomly assigned usual a diet with advice to reduce fats (control group) or a Mediterranean diet (MeDiet) supplemented with either extra virgin olive oil (1 L/week) or mixed nuts (30 g/die).

Regarding omega-3, the LC PUFAs’ target exposure was set to the recommendation for primary cardiovascular protection (500 mg/die), a goal that could be achieved by consuming two weekly servings of fish; meeting the recommendations was assessed via validated food frequency questionnaires that included eight items regarding seafood products.

Among the 3482 patients, during a median follow-up period of 6 years, 69 patients with type 2 diabetes developed new sight-threatening retinopathy, defined as requiring laser photocoagulation, intravitreal anti-VEGF injection, and/or vitreoretinal surgery.

After adjusting for classic risk factors and the interventional group, patients reporting intake of at least 500 mg/die of DHA + EPA at baseline showed a significantly reduced risk of developing sight-threatening DR, amounting to 46%; these results remained significant after several sensitivity analyses [23].

A 2-year randomized, double-blind, placebo-controlled study was designed to assess the effect of high dose DHA supplementation in the progression of diabetic retinopathy in diabetic patients with the non-proliferative form of the disease [24]. On the whole, 170 patients with diabetes were randomly assigned to either an omega-3 PUFA-rich triglyceride supplementation (total of 1050 mg PUFAs/die, including 350 mg DHA and 42.5 mg EPA) or olive oil capsules (placebo group).

Considering a temporal frame of 2 years, no significant difference in slowing the progression of the disease or visual acuity was reported.

A prospective controlled study was designed to evaluate macular function by fundus microperimetry in asymptomatic patients with non-proliferative diabetic retinopathy who were assigned to receiving either DHA supplementation (comprised of the same supplement mentioned in the previous study) or placebo capsules [25].

The study recruited 24 patients randomized in a 1:1 fashion; assessment after 90 days of the beginning of the intervention revealed significantly better macular sensitivity and integrity in the interventional group compared to the placebo group.

### 2.3. Retinopathy of Prematurity

Regarding the possible role of omega-3 in the development of the retina, several clinical trials have evaluated the possibility to prevent the occurrence of retinopathy of prematurity (ROP) in preterm infants either before or after birth by the supplementation of omega-3 PUFAs. The Mega Donna Trial [26] was a randomized, clinical trial designed to assess whether enteral supplementation with fatty acids prolonged from birth until 40 weeks postmenstrual age could reduce the incidence of ROP in extremely preterm infants.

The trial was preceded by a cohort study aimed at assessing whether the severity of ROP was associated with the serum levels of LC-PUFAs, particularly DHA and arachidonic acid (AA) [27]. By recruiting a total of 171 infants, it revealed that a higher mean daily serum level of DHA during the first 28 postnatal days was associated with less severe forms of ROP, even after adjustment other for known risk factor, but only in the presence of sufficiently high levels of arachidonic acid.

The clinical trial enrolled a total of 101 infants receiving the enteral supplementation (AA:DHA group) with arachidonic acid (AA, 100 mg/kg/die) and DHA (50 mg/kg/die), in a time period going from within 3 days after birth until 40 weeks’ postmenstrual age, while 105 infants received no supplementation. The study results provided compelling results in showing that the enteral AA:DHA group presented with a 50% reduced risk of developing ROP compared to no supplementation, and these patients had significantly higher serum levels of both AA and DHA.

A non-parallel group cohort study recruited infants born before 33 weeks of gestational age or with a birth weight ≤1500 g; among 155 patients, 81 were in the control group, while 74 received oral oil drops as DHA supplementation (66.3 mg per drop dosed based on the infant’s weight) [28]. A higher but non-significant incidence of ROP was observed in patients not receiving the supplementation. Unadjusted logistic regression showed a significant association of patent ductus arteriosus and neonatal corticosteroids with ROP in both groups; this remained significant in the unsupplemented group after adjustment, while only surfactant use remained significantly associated with ROP in the DHA group.

Another double-blind, parallel clinical trial recruited preterm infants with birth weight <1500 g and ≥1000 g but in a neonatal intensive care unit; they were randomized to receive either 75 mg of DHA/kg/die or high oleic sunflower oil (control group) [29]. No significant difference was observed for general risk of developing ROP; however, the risk of stage 3 ROP was significantly lower in the active group.

## 3. Dosage and Safety

Supplemental intakes of EPA and DHA at doses of 2–6 g/day and DHA at doses of 2–4 g/day yield an approximate 3% elevation in LDL cholesterol levels, without adversely affecting cardiovascular disease risk [30]. The administration of EPA at doses up to 4 g/day does not appreciably affect LDL cholesterol levels. Supplemental doses of EPA and DHA together at amounts up to 5 g/day, and supplemental intakes of EPA alone up to 1.8 g/day, are safe for adults. Dietary recommendations for EPA and DHA, concerning cardiovascular risk for European adults, range from 250 to 500 mg daily. The supplementation of DHA alone at doses nearing 1 g/day is deemed safe for the general populace. The consumption of EPA and DHA has not been linked to negative effects in healthy adults or children at the observed intake levels. As long as the oxidative stability of omega-3 fatty acids is preserved, the extended supplementation of EPA and DHA at doses nearing 5 g/day does not seem to elevate the risk of spontaneous bleeding events or their repercussions, nor does it influence glucose homeostasis, immune responses, or lipid peroxidation.

Observational studies were conducted to evaluate the effectiveness of EPA and DHA in age-related macular degeneration. In those studies, the median intake of EPA or DHA varied from 200 to 350 mg/day for individuals in the highest consumption category, but our study indicated a range of 400 to 600 mg/day. In contrast, two double-blind, placebo-controlled, randomized trials, the AREDS2 study conducted in the United States and the Nutritional AMD Treatment 2 study (NAT2) from France, found that the high-dose supplementation of EPA and DHA (AREDS2: 350 mg/day DHA and 650 mg/day EPA; NAT2: 840 mg/day DHA and 270 mg/day EPA) did not reduce the risk of progression to advanced AMD over a follow-up period of 3 to 5 years.

Confounding may explain inverse associations in observational studies, while several factors could clarify the null results in randomized trials, including insufficient follow-up duration, intervention timing that did not capture the true latent period, inadequate compliance (NAT2 trial), and elevated baseline intakes of EPA and DHA. In this study, although the pooled analysis revealed no connections with advanced AMD, the HPFS demonstrated substantial inverse linkages, particularly when utilizing the projected biomarker scores.

## 4. Anti-Angiogenic Properties

Angiogenesis is the formation of new blood vessels from existing ones. This has been a notable reported prognostic outcome in multiple retinal disorders, including age-related macular degeneration (AMD), diabetic retinopathy (DR) [31], and the retinopathy of prematurity (ROP), as it leads to complications, such as vitreous hemorrhages and macular edema due to brittle and undeveloped vessels. Vascular endothelial growth factor (VEGF) is a vital mediator that promotes enhanced endothelial permeability and retinal neovascularization.

Matrix metalloproteinases (MMPs) are crucial to both normal and pathological angiogenesis through the modulation of cellular signaling and tissue remodeling. The degradation of the basement membrane promotes the migration and proliferation of endothelial cells, actions governed by MMPs [32]. Additionally, the COX-2 production of pro-inflammatory eicosanoids enhances VEGF expression. VEGF, MMPs, and COX-2 are together crucial in the inflammatory angiogenesis that drives the progression of diabetic retinopathy [33].

Matesanz et al. investigated the influence of omega-3 PUFAs on angiogenic signaling. Primary retinal microvascular endothelial cells (RMECs) derived from bovine eyes were exposed to DHA or EPA for 48 h. The migration of RMECs was assessed using a scratch-wound test, proliferation was measured via BrdU incorporation, and angiogenic sprouting was investigated through a three-dimensional in vitro angiogenesis model. The DHA therapy resulted in reduced superoxide production and attenuated the response to VEGF-induced superoxide and nitrite release, significantly impairing endothelial wound healing, proliferation, and angiogenic sprout development. DHA enhances NO bioavailability, decreases superoxide production, and attenuates VEGF-mediated angiogenic signaling. The results indicate that omega-3 polyunsaturated fatty acids, particularly DHA, are crucial for preserving vascular integrity and mitigating pathogenic retinal neovascularization [34,35].

DHA demonstrated a more pronounced elevation in NO and a corresponding decrease in O_2_ regarding their redox balancing capacities relative to EPA. Strategies aimed at decreasing O_2_ levels or increasing NO bioavailability can facilitate reparative angiogenesis [36]. These data collectively suggest that DHA may have therapeutic advantages over EPA in treating vasoproliferative disorders. A possible reason for this differential effect may relate to the presence of an additional unsaturated double bond in DHA, which would increase the unsaturation index compared to EPA. Furthermore, DHA has exhibited enhanced anti-inflammatory effectiveness and has prompted greater vasodilation in comparison to EPA [37,38,39]. These findings support prior research indicating that EPA and DHA reduced serum-induced endothelial cell migration, proliferation, and tube formation. Yang et al. [40] performed a study in which bovine carotid artery endothelial cells were subjected to 0–5 mg/mL of EPA for 48 h, resulting in a dose-dependent inhibition of VEGF (0.2 nM)-induced proliferation.

Fernando et al. investigated the in vitro, ex vivo, and in vivo anti-angiogenic effects of phloridzin docosahexaenoate (PZ-DHA), a new ω-3 fatty acid ester generated from a flavonoid precursor. This molecule combines phloridzin (PZ), a flavonoid precursor derived from apple peels, with DHA through an enzyme-catalyzed acylation process. Human umbilical vein endothelial cells (HUVEC) and human dermal microvascular endothelial cells (HMVEC) subjected to a sub-cytotoxic dose of PZ-DHA exhibited impaired tubule formation on a Matrigel matrix, signifying in vitro anti-angiogenic activity. Ex vivo angiogenesis was evaluated using rat thoracic aortas, revealing reduced arterial sprouting and tubule formation in the presence of PZ-DHA. Female BALB/c mice implanted with Matrigel plugs containing VEGF165 and basic fibroblast growth factor demonstrated a significant reduction in angiogenesis following PZ-DHA treatment. PZ-DHA inhibited the proliferation of HUVEC and HMVEC, as well as the migration of HUVECs in gap closure and transwell cell migration assays [41].

The AREDS and AREDS2 cohort studies indicated a significant association between DHA and omega-3 fatty acid intake and a reduced incidence of neovascular age-related macular degeneration (nAMD) (Agrón). A randomized controlled trial conducted by Souied et al. [42] revealed that three years of oral DHA supplementation did not significantly affect the occurrence of choroidal neovascularization (CNV) in the contralateral eye of patients with unilateral neovascular age-related macular degeneration (nAMD).

The VITAL experiment demonstrated that marine omega-3 fatty acid supplementation had no significant impact on the incidence or progression of AMD. The data suggest that, while potential connections exist between omega-3 consumption and AMD risk, the effect of supplementation may be less pronounced in some patient categories [43].

## 5. Neuroprotective Effects

Increased dietary consumption of ω-3 PUFAs has been shown to provide significant benefits to retinal health, enhancing the function of photoreceptors and bipolar cells, with the most notable improvements seen in retinal ganglion cell (RGC) function, according to Nguyen et al. [44,45]. Additionally, further research has demonstrated that ω-3 PUFA supplementation offers protective effects against injuries to both the brain and retina [46,47,48,49].

Kalogerou et al. examined the neuroprotective benefits of omega-3 polyunsaturated fatty acid (ω-3 PUFA) supplementation in a murine model of OPA1-associated autosomal dominant ocular atrophy (ADOA). Eight-month-old mice were allocated into treatment and control groups and administered daily dosages of EPA and DHA for four months, according to designated blood–fatty acid ratios. The results indicated elevated EPA and reduced arachidonic acid levels in the blood and retina, resulting in enhanced retinal ganglion cell and optic nerve axonal densities in the treated mice relative to the controls. Moreover, the treated groups exhibited a diminished number of apoptotic cells, decreased inflammation, and reduced levels of pro-apoptotic markers. The results indicate that ω-3 PUFAs may confer neuroprotection via suppressing inflammatory responses rather than mitigating oxidative stress, underscoring their potential as a treatment for ADOA and warranting more clinical trials [49].

In this study, mice treated with ω-3 PUFAs exhibited microglia with a ramified and quiescent morphology, characterized by longer and thinner processes compared to untreated mice, which had shorter and thicker microglial processes. While a balanced diet of ω-6 and ω-3 PUFAs did not prevent diabetes-induced changes in Müller cell processes [50], two recent clinical trials indicated that oral ω-3 PUFA supplements can help lower the levels of pro-inflammatory cytokines associated with discomfort from contact lenses and dry eye disease [51,52].

More specifically, a diet high in alpha-linolenic acid (ALA), which includes 50-60% of its content from sources such as flaxseeds, walnuts, leafy greens, soybeans, and certain oils, can provide substantial health benefits [53]. ALA has been shown to possess cytoprotective and neuroprotective properties, notably reducing inflammatory mediators, such as VEGF and pro-inflammatory cytokines (IL-6, IL-1β, TNF-α), in diabetic retinopathy models [54]. Conjugated isomers of ALA help mitigate oxidative stress by lowering the activity of nitric oxide synthase (NOS), reducing lipid peroxidation, and protecting DNA from damage, while enhancing the function of antioxidant enzymes. As demonstrated in a human study, ALA also promotes the production of brain-derived neurotrophic factor (BDNF), a protein that safeguards neurons from damage and supports their survival, thus preventing retinal degeneration [55].

Furthermore, a derivative of ALA, 13-hydroxyoctadecadienoic acid, has been shown to inhibit the expression of several MMPs associated with inflammation and angiogenesis in obese models [56]. Overall, ALA demonstrates protective effects against diabetes-induced changes, suggesting its potential to prevent the progression of diabetic retinopathy [31]. Notwithstanding, DHA has been studied for its protective effects in the early stages of both Alzheimer’s disease (AD) and Parkinson’s disease (PD). The addition of DHA on cultured Wistar rat retinas exposed to oxidative stress from paraquat resulted in reduced photoreceptor apoptosis and maintained mitochondrial membrane integrity. Furthermore, DHA treatment increased the expression of the anti-apoptotic protein Bcl-2 [57]. Conversely, in SD rats fed with linolenic acid, a precursor to DHA, the accumulation of DHA in photoreceptors led to greater damage to retinal and RPE cells following light exposure compared to control animals. This suggests that, while DHA has protective qualities, it is also susceptible to oxidation, which may contribute to retinal damage under certain conditions [58]. The neuroprotective effects of EPA and DHA and their derivatives are summarized in Table 1 below.

## 6. Population Studies

Several population studies have been conducted thus far, evaluating baseline omega-3 PUFAs levels and the effect of their dietary implementation, in real patient cohorts.

### 6.1. Age-Related Macular Degeneration

Few studies have examined data from the National Health and Nutrition Examination Study (NHANES) cohort conducted between 2005 and 2008, which is a population-based cross-sectional study representative of the general US population [59,60].

In a cross-sectional study involving patients aged over 40, 4702 individuals were included, of whom 374 were diagnosed with age-related macular degeneration (AMD). Specifically, 328 participants had early AMD, characterized by the presence of drusen or pigmentary abnormalities, while 46 exhibited late AMD, marked by signs of exudation or geographic atrophy. Dietary fatty acid intake was evaluated through two 24 h dietary recall interviews [59].

Upon controlling for pertinent covariates, each unit (1 mg/1000 kcal) increase in EPA and DHA intake was associated with reduced risks of AMD. The greatest and lowest quartiles of EPA, DPA, and DHA exhibited a negative association with any manifestation of AMD.

Increased dietary intake of DHA, DPA, and DHA correlated with reduced risks of early AMD; however, no definitive link between late AMD and any fatty acids was seen.

A separate cross-sectional study conducted on the same cohort, involving 4842 participants, corroborated the strong inverse relationship between EPA and DHA intake and AMD risk, while indicating a possible influence of age, education level, BMI, and history of cataract surgery on this correlation.

An examination of a US cohort of post-menopausal women from the Women’s Health Initiative (WHI) Clinical Trials assessed the correlation between red blood cell (fasting serum samples) and dietary fatty acids with prevalent and incident age-related macular degeneration (AMD).

Of the 1456 postmenopausal women studied, 240 had existing age-related macular degeneration (AMD), and 138 reported the onset of AMD over a 9.5-year period. No significant correlation was found between serum and dietary eicosapentaenoic acid (EPA) and docosahexaenoic acid (DHA) and AMD; however, dietary consumption of arachidonic acid (AA) and the ratio of linoleic acid to alpha-linolenic acid indicated a trend towards increased risk [60].

A cross-sectional, case-control study assessing plasma fatty acid levels in relation to neovascular age-related macular degeneration (AMD) in a Chinese cohort, comprising 99 patients with neovascular AMD and 198 controls, reported that reduced circulatory levels of DHA and EPA, along with omega-6 polyunsaturated fatty acids (PUFAs), were associated with neovascular AMD [45].

A recent meta-analysis of observational cohort studies revealed significant findings: the dietary consumption of EPA and DHA was inversely correlated with early age-related macular degeneration (AMD), with each 1 g/day increase in DHA and EPA linked to a 60% and 50% reduction, respectively; additionally, plasma levels of DHA and EPA indicated a markedly inverse relationship with advanced AMD [61,62].

### 6.2. Diabetic Retinopathy

Another study based on the NHANES 2005–2008 survey cohort evaluated specifically the incidence of diabetic retinopathy in relation to omega-3 intake, in patients over 40 years of age with a history of diabetes.

Among the 1243 patients meeting inclusion criteria, omega-3 intake showed significant and negative association with DR incidence; more specifically, patients with a higher DHA and docosapentanoid acid (DPA), but not EPA intake, were significantly less at risk of developing DR [63].

A combined analysis from patients belonging to the Multi-Ethnic Study of Atherosclerosis (MESA) and the Genetics of Latino Diabetic Retinopathy (GOLDR) cohorts provided intriguing results [64].

Considering the combined population of 1356 eligible individuals with type 2 diabetes, individuals in the fourth quartile of plasmatic DHA had a significantly lower risk (17% less) of developing retinopathy as compared with the first quartile. Furthermore, secondary analysis revealed that the same patients had also a 37% significantly lower severity when compared with the first DHA quartile and with the first EPA + DHA quartile. No significant association was observed between EPA and retinopathy. A summary of the findings is represented in Table 2.

## 7. Combination with Other Nutrients

The Age-Related Eye Disease Study 2 (AREDS 2) evaluated the effect of the supplementation of omega-3 (DHA and EPA) and lutein/zeaxanthin against the original antioxidant nutrients combination used in the AREDS 1 (vitamin E 400 UI, vitamin c 500 mg, β-carotene 15 mg, zinc oxide 80 mg, cupric oxide 2 mg) in patients with AMD [16,66]. Vitamin C, vitamin E, and zinc are known for their antioxidative properties when delivered at high dosages; nevertheless, collateral effects like lung carcinoma (vitamin E) and drusen formation (zinc) must be avoided [20]. The results showed that the addition of such elements did not improve the outcomes and the risk of progression to advanced AMD [1].

A research group evaluated the benefits, on a group with unilateral exudative AMD, of a dietary supplementation based on the AREDS compounds, omega-3, resveratrol, and hydroxytyrosol compared to the AREDS, manganese, and selenium combination. Interestingly, the first group did not show, at 12 months, a significantly different visual improvement, making omega-3 supplementation a less promising therapy for AMD [67].

A study conducted in 2021 evaluated the effect of different combinations of omega-3, antioxidants, and resveratrol [66]. Resveratrol is known for his protective properties against light-induced retinal damage [68]. They delivered Resvega (a combination of omega-3 and resveratrol called RSG or RGA) using the same recommendations of the AREDS, Nutrof (NUT) containing only omega-3 fatty acids, and resveratrol alone (RSV), demonstrating the protective effects on CNV formation, especially with the RSG formulation rather than RSV or omega-3 alone [66,69]. The protection against CNV development is probably mediated by the reduced levels of VEGF related to the interaction between the VEGF-receptor (VEGF-R) and caveolin-1 (CAV-1) [69,70]. NUT properties are also directed to the glutamate/glutamine cycle providing a protection to Muller cells from oxidative stress, representing a potential treatment in light-induced retinopathies [71].

The addition of lutein and zeaxanthin to omega-3 in a formulation called LUTEGA showed an augmentation of pigment density in patients with non-neovascular AMD [20]. Another study conducted on mice with a retinal degeneration with AMD-like features compared the effects of a diet based on xanthophyll and omega-3 PUFAs and a control isocaloric diet. According to the results, such supplementation would reduce the levels of iNOS, TNF-α, IL-1β, VEGF, and COX-2 but also preserve some structural features of the retina and EPR [72].

## 8. Conclusions

In conclusion, eicosapentaenoic acid (EPA) and docosahexaenoic acid (DHA) have demonstrated significant potential in the preventive treatment of oxidative stress in retinopathy. The anti-inflammatory and antioxidant properties of these omega-3 fatty acids play a crucial role in mitigating the pathological processes associated with oxidative stress, which is a key contributor to retinal damage and the progression of retinopathy. Studies have shown that EPA and DHA can enhance cellular resilience against oxidative damage, reduce inflammation, and support retinal health by modulating various signaling pathways involved in cell survival and repair. EPA and DHA demonstrate considerable efficacy in alleviating oxidative stress associated with retinal diseases. Their incorporation into preventive and therapeutic measures may be crucial for safeguarding eyesight and alleviating the impact of retinopathy. Additional research is crucial to enhance their utilization and comprehend their long-term effects.

Furthermore, the inclusion of EPA and DHA in dietary interventions has been associated with improved visual outcomes and a decrease in the incidence and severity of retinopathic conditions. While the current body of research is promising, continued clinical studies and trials are essential to fully understand the optimal dosages, mechanisms of action, and long-term effects of these fatty acids in the context of retinal health. Ultimately, the integration of EPA and DHA into preventive strategies offers a promising avenue for reducing the burden of retinopathy and preserving vision, highlighting the need for further exploration and implementation in clinical practice.

## Figures and Tables

**Table 1:** Summary of findings on the effect of EPA, DHA, and ALA on retinal health.

Study/Source	Subjects	Omega-3 Fatty Acids Studied	Key Findings	Effects on Retinal Health
Nguyen et al. [44,45]	Human and animal models	ω-3 PUFAs (EPA, DHA)	Significant benefits to retinal health, improving photoreceptor and bipolar cell function. Notable improvements in retinal ganglion cell (RGC) function.	Enhances RGC function, photoreceptor, and bipolar cell function.
Kalogerou et al. [49]	Murine model (OPA1-associated ADOA)	EPA, DHA	EPA supplementation reduced arachidonic acid levels and improved retinal ganglion cell density and optic nerve axon density. Reduced apoptotic cells, inflammation, and pro-apoptotic markers.	Neuroprotection through suppression of inflammation. Improved retinal structure and function.
Yee et al. [50]	Mice	ω-3 PUFAs	Balanced ω-6/ω-3 diet did not prevent diabetes-induced changes in Müller cell processes.	No effect on Müller cell processes in diabetic mice.
Deinema et al. [51], Downie et al. [52]	Human subjects (contact lens users, dry eye disease)	ω-3 PUFAs	Oral ω-3 PUFA supplements reduced pro-inflammatory cytokines, alleviating discomfort from contact lenses and dry eye disease.	Reduces inflammation in dry eye disease and contact lens discomfort.
De Lorgeril et al. [53]	Human subjects, dietary in-take	ALA (flaxseeds, walnuts, etc.)	High ALA intake demonstrated neuroprotective and cytoprotective properties and reduced inflammatory mediators (VEGF, IL-6, IL-1β, TNF-α).	Reduces inflammatory mediators in diabetic retinopathy models, neuroprotective properties.
Shen et al. [54]	Animal models (diabetic retinopathy)	ALA	ALA-mitigated oxidative stress by lowering nitric oxide synthase activity and lipid peroxidation. Enhanced antioxidant enzyme function.	Reduces oxidative stress, protecting against retinal degeneration in diabetic retinopathy.
Hadjighassem et al. [55]	Human subjects	ALA	ALA promotes production of BDNF, a protein protecting neurons and supporting survival.	Protects against retinal degeneration, promotes neuronal survival.
Caliguiri et al. [56]	Obese murine models	13-hydroxyoctadecadienoic acid (ALA derivative)	ALA derivative inhibits MMP expression, reducing inflammation and angiogenesis.	Prevents retinal damage and inflammation in obese models.
Rotstein et al. [57]	Cultured rat retinas	DHA	DHA reduced photoreceptor apoptosis, maintained mitochondrial membrane integrity, increased Bcl-2 expression.	Protects photoreceptors from oxidative stress.
Organisciak et al. [58]	SD rats	DHA	DHA accumulation in photoreceptors resulted in greater retinal damage after light exposure, suggesting DHA oxidation could contribute to damage.	DHA has protective qualities but may contribute to retinal damage under oxidative conditions.

**Table 2:** Large scale human studies on ω-3 PUFAs.

Study	Population	Sample Size	Age Range	Key Findings	Omega-3 Fatty Acids Studied	Outcome/Association
NHANES (2005–2008) Cross-Sectional Study [65]	US population, aged 40+	4702 individuals	40+	− 374 diagnosed with AMD; 328 with early AMD, 46 with late AMD. − Increased intake of EPA, DHA, DPA correlated with reduced risk of early AMD.	EPA, DHA, DPA	Negative association between increased EPA/DHA intake and early AMD. No definitive link with late AMD.
WHI Clinical Trial (Post-Menopausal Women) [60]	Post-menopausal women	1456 women	Post-menopausal	− 240 had existing AMD; 138 developed new AMD over 9.5 years. − No significant correlation between EPA/DHA intake and AMD.	EPA, DHA, AA, LA/ALA ratio	No significant link between serum or dietary EPA/DHA and AMD; potential increased risk with AA and LA/ALA ratio.
Chinese Cohort Case-Control Study [45]	Chinese cohort	297 individuals (99 with neovascular AMD, 198 controls)	N/A	− Reduced circulatory levels of DHA, EPA, and omega-6 PUFAs linked to neovascular AMD.	DHA, EPA, Omega-6 PUFAs	Negative association between DHA, EPA, and neovascular AMD.
Meta-Analysis of Observational Cohorts [61,62]	Various populations	Multiple cohorts	Various	− Inverse correlation between DHA/EPA intake and early AMD. − Each 1 g/day increase in DHA reduced early AMD risk by 60%, EPA by 50%.	DHA, EPA	Inverse relationship between DHA/EPA intake and early AMD; plasma DHA/EPA also inversely correlated with advanced AMD.
NHANES (2005–2008) Diabetic Retinopathy Study [63]	US population, with diabetes	1243 individuals	40+	− Higher DHA and DPA intake, but not EPA, significantly reduced risk of diabetic retinopathy (DR).	DHA, DPA, EPA	DHA/DPA intake negatively correlated with DR incidence.
MESA and GOLDR Cohorts Combined Analysis [64]	Individuals with type 2 diabetes	1356 individuals	N/A	− Increased DHA levels associated with a 17% reduced risk of DR. − Higher DHA also correlated with 37% lower severity of DR.	DHA, EPA	Increased DHA linked to reduced risk and severity of DR. No significant correlation with EPA.

N/A: not applicable.
